# Comparison of Treatment Options of Three- and Four-Part Humerus Proximal Fractures in Patients Over 50 Years of Age

**DOI:** 10.7759/cureus.17516

**Published:** 2021-08-28

**Authors:** Firat Erpala, Mesut Tahta, Tahir Öztürk, Çağatay Zengin

**Affiliations:** 1 Orthopedics and Traumatology, Cesme State Hospital, Izmir, TUR; 2 Orthopedics and Traumatology, Egepol Surgery Hospital, Izmir, TUR; 3 Orthopedics and Traumatology, Tokat Gaziosmanpaşa University, Tokat, TUR; 4 Orthopedics and Traumatology, Gaziosmanpasa University School of Medicine, Tokat, TUR

**Keywords:** proximal humerus fractures, shoulder hemiarthroplasty, osteosynthesis, neer 3- and 4-part fractures, upper extremity trauma

## Abstract

Introduction

Our aim was to evaluate different treatment methods including conservative treatment (CT), locking-plate osteosynthesis (OS) and hemiarthroplasty (HA) in three- and four-part humeral fractures in patients older than 50 years.

Methods

Forty-seven patients that have at least one year of follow-up were divided into three groups: 18 patients treated with OS, 14 patients treated conservatively and 15 patients treated with HA. For further evaluation, constant shoulder score, disabilities of the arm, shoulder and hand score (DASH), American Shoulder and Elbow Society (ASES) score had been used. Shoulder range of motion was also assessed.

Results

OS and CT groups had better scores than HA group. In OS group, average Constant score was 71.6 ± 16.2, DASH score was 12.1 (5.2-24.2) and ASES score was 77.5 (50.8-96.6). In CT group, average Constant score was 69.6 ± 19.2, DASH score was 16.4 (12.5-36.7) and ASES score was 76.6 (45.4-87.9). DASH scores (p = 0.032), Constant scores (p = 0.001), forward elevation (p < 0.001), abduction (p < 0.001), internal (p = 0.022) and external rotation (p = 0.048) were significantly improved in OS and CT groups than HA group.

Conclusions

HA should not be considered a priority in surgical planning in Neer three-part and four-part proximal humerus fractures. CT is superior for patients with additional morbidity and advanced age. But in patients who are younger and can tolerate the surgical procedure, the priority should be OS.

## Introduction

Proximal humerus fractures account for between 3.7% and 10% of all fractures [1]. Parallel to the increase in the geriatric population, there is an increase in the incidence of proximal humerus fractures seen in advanced ages [2]. After proximal femur and distal radius fractures, proximal humerus fractures are the third most common fracture in geriatric females [3-4]. After 40 years of age, bone mineral concentrations and vitamin D activities decrease in both males and females and this is obvious in women after menopause [5,6]. Two-thirds of women and one-fifth of men 50 years and older are at elevated osteoporotic fracture risk [7].

Considering the patient's age and accompanying diseases, the appropriate treatment can be selected from options such as conservative approach, osteosynthesis with plate screw, fixation with Kirschner wire, osteosynthesis with intramedullary nail, partial and total shoulder arthroplasty in multi-part proximal humerus fractures [8-10]. Although different treatment strategies have been tried to be popularized in various studies in the literature, the evaluation and management of these injuries in elderly patients is often controversial and challenging [11-13].

The aim of this study was to evaluate the clinical results of conservative treatment, locking plate osteosynthesis and hemiarthroplasty approaches, and subsequent shoulder function for patients with Neer Type III and Type IV fractures.

## Materials and methods

Patients who were admitted to the emergency department and outpatient clinics with a diagnosis of proximal humerus fracture between 2009 and 2019 in the orthopaedics and traumatology clinic were retrospectively evaluated for the study. The study was carried out in accordance with the Declaration of Helsinki, and approval was obtained from the ethics committee of Izmir Katip Celebi University Atatürk Research Hospital (Ethics committee date; 21.04.2016, Institutional Board Number; 96).

Among these patients, those with Type III and Type IV fractures according to the Neer classification who were 50 years or older were identified, and patients with a minimum follow-up of one year were included in the study. Patients with pathological fractures, open fractures, neuromuscular diseases, cognitive dysfunction, history of stroke, hemiplegia, those who had undergone surgery with other surgical techniques and those who did not want to participate were excluded.

The conservative treatment group was determined as patients who could not have surgical planning due to comorbid factors and patients who refused the operation at their own will. According to the American Society of Anesthesiologists (ASA) physical status scale, nine patients were ASA 3 and five patients were ASA 4. Primarily osteosynthesis was planned in all patients who underwent surgical intervention. Simultaneously hemiarthroplasty was planned for every fracture that underwent surgery if needed. Hemiarthroplasty was considered for irreducible comminuted fractures of joint surface, split fractures of humeral head with high risk of avascular necrosis and irreducible fractures that include collum anatomicum.

A total of 47 patients met the criteria and were divided into three groups: locking plate osteosynthesis (Group I; n = 18), hemiarthroplasty (Group II; n = 15) and conservative treatment (Group III; n = 14). Patients were evaluated using Constant Shoulder Scoring [14], American Shoulder and Elbow Surgeons Scoring (ASES) [15], and Disabilities of the Arm, Shoulder and Hand (DASH) [16] scoring at the last follow-up. Shoulder joint range of motion was compared using a goniometer.

In addition to shoulder antero-posterior and lateral X-rays, computed tomography was performed in emergency and outpatient clinic applications. In all groups, patients met the criteria for surgical intervention (more than 45° of articular surface angulation and more than 1 cm displacement of fracture parts) cited by Neer [17].

Surgical technique

Patients undergoing locking plate osteosynthesis and hemiarthroplasty were prepared in the beach chair position, and a deltopectoral incision was made. Imaging was performed during surgery using a C-arm scope. In group I, 10-12 cm long skin incision between the coracoid process and the proximal humeral shaft was performed. Deltopectoral groove with the cephalic vein was exposed. Deltoid muscle retracted laterally and under clavipectoral fascia, proximal humerus was exposed. After reduction, the plate was placed lateral to bicipital groove and pectoralis major tendon, 5-10 mm distal to the superior edge of the greater tuberosity (Proximal Humerus Locking Plates TI, TST Medical Devices, Pendik/Istanbul, Turkey). Especially inferomedial calcar screws were placed to prevent varus collapse. Subscapularis tenotomy was performed to maintain anatomical reduction if needed. In group II same exposure was used. After following landmarks, proximal humerus was assessed for metaphyseal comminution and shortening. Humeral preparation and reaming were done according to 30 degrees retroversion. After appropriate head and stem size was chosen and prosthesis placement, greater tuberosity was reconstructed with wires and nonabsorbable sutures if needed (SMR Shoulder Systems®, Lima Corporate, Villanova, Italy).

Postoperative follow-up

**Figure 1:**
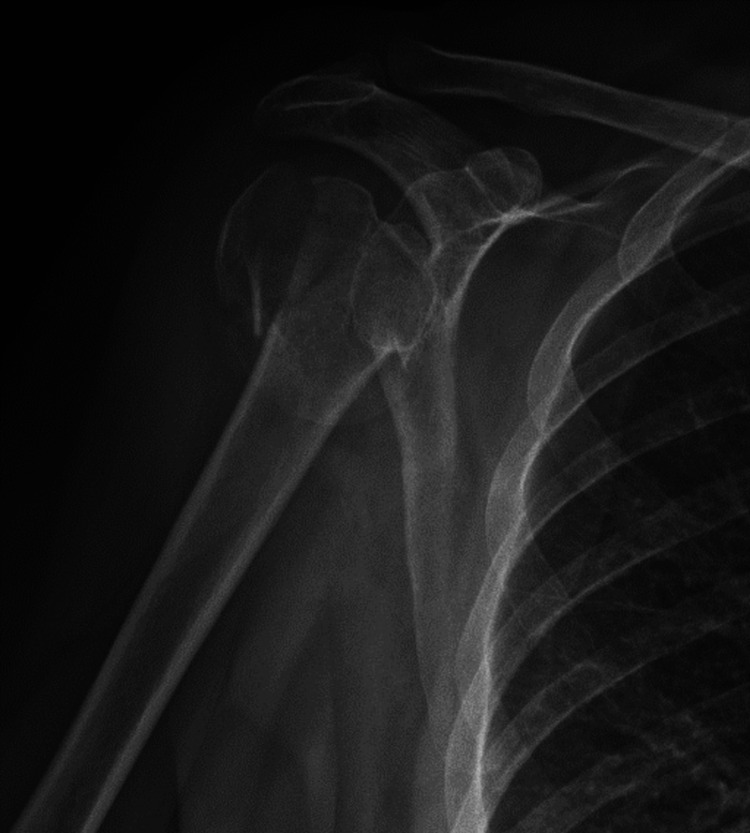
A 61-year-old female’s preoperative X-ray.

**Figure 2:**
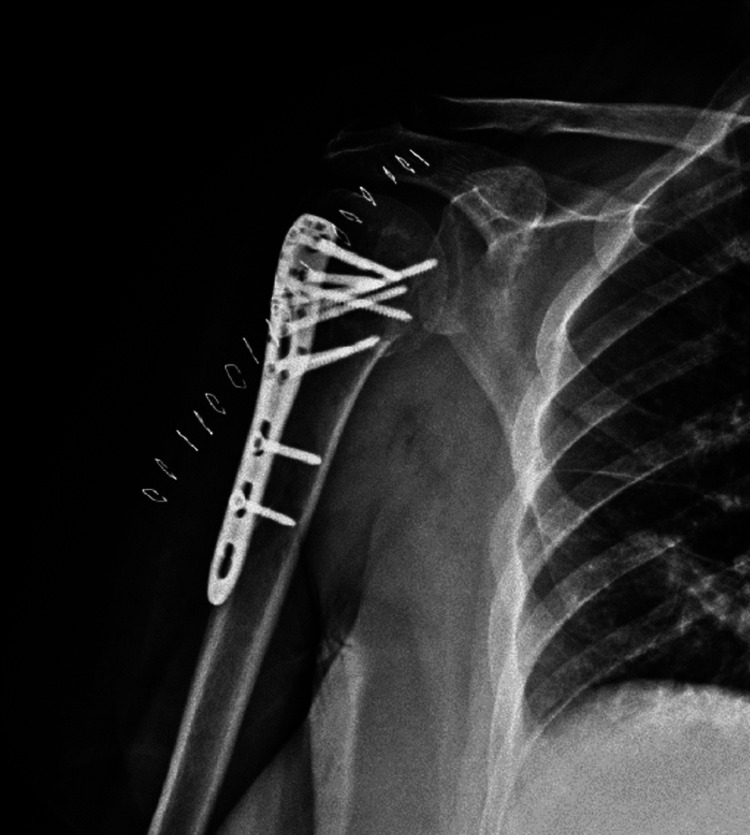
Locking plate osteosynthesis postoperative X-ray.

Following surgery, patients in group I were followed up in a sling for two weeks. Passive shoulder exercises with the help of a clinical physiotherapist were performed in the second week, followed by active assisted exercises in the fourth week and strengthening exercises after the sixth week (Figures 1, 2).

**Figure 3:**
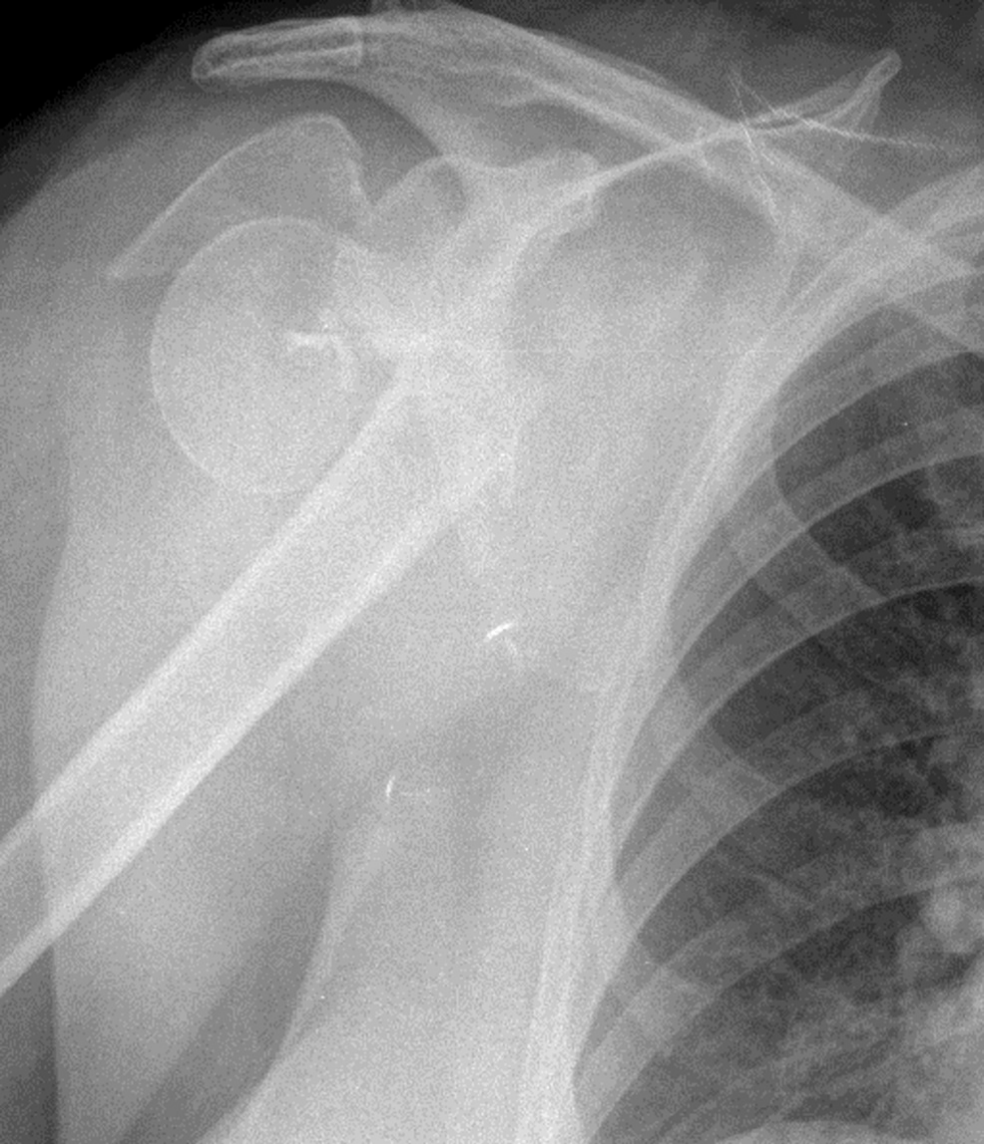
A 75-year-old female’s preoperative X-ray.

**Figure 4:**
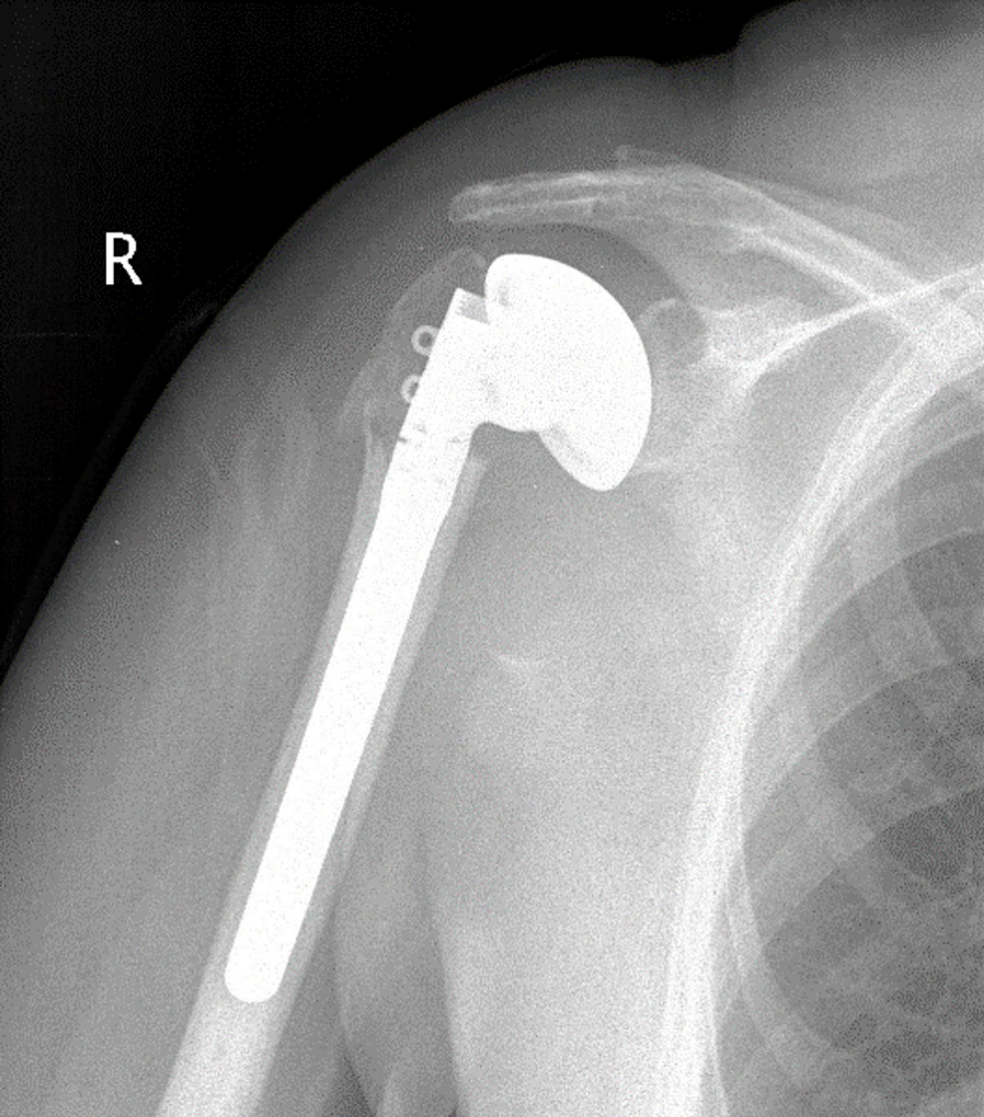
Postoperative X-ray after hemiarthroplasty (HA).

Patients in group II were assisted by a physiotherapist in performing passive shoulder exercises in the first week, active-assisted exercises in the third week and strengthening exercises in the sixth week (Figures 3, 4).

**Figure 5:**
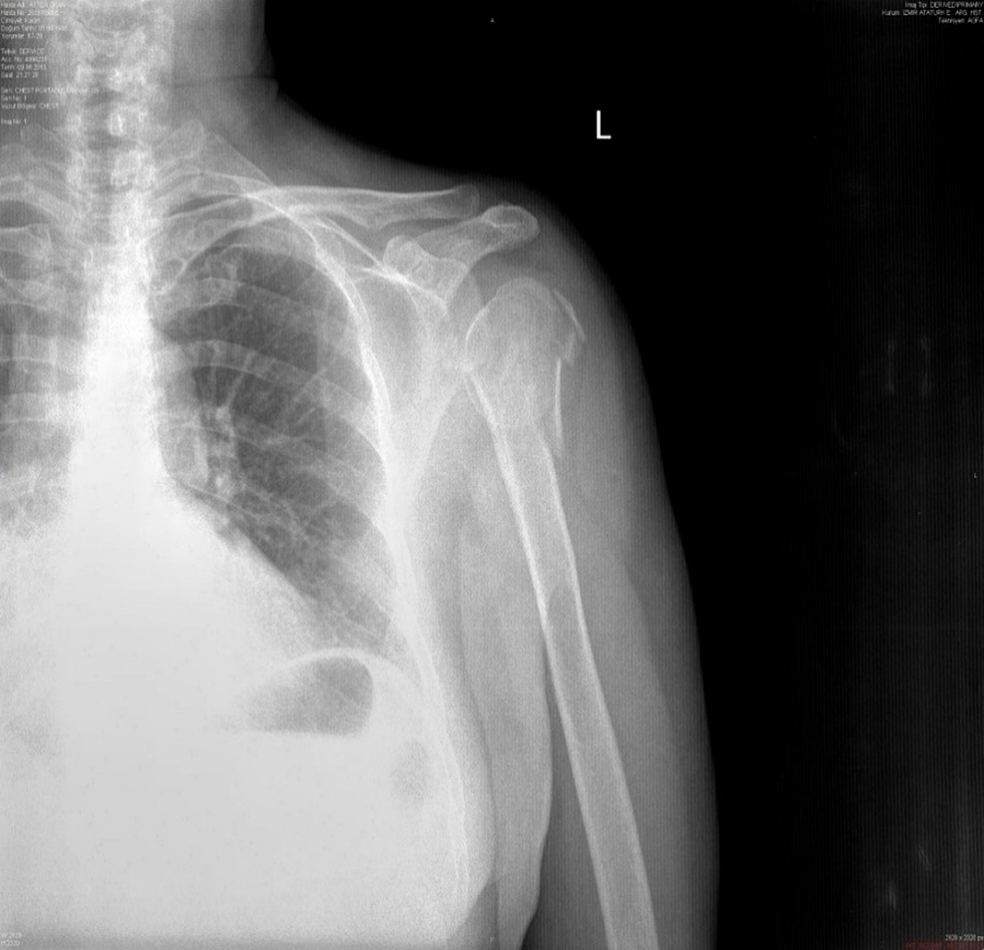
A 70-year-old female’s first X-ray.

**Figure 6:**
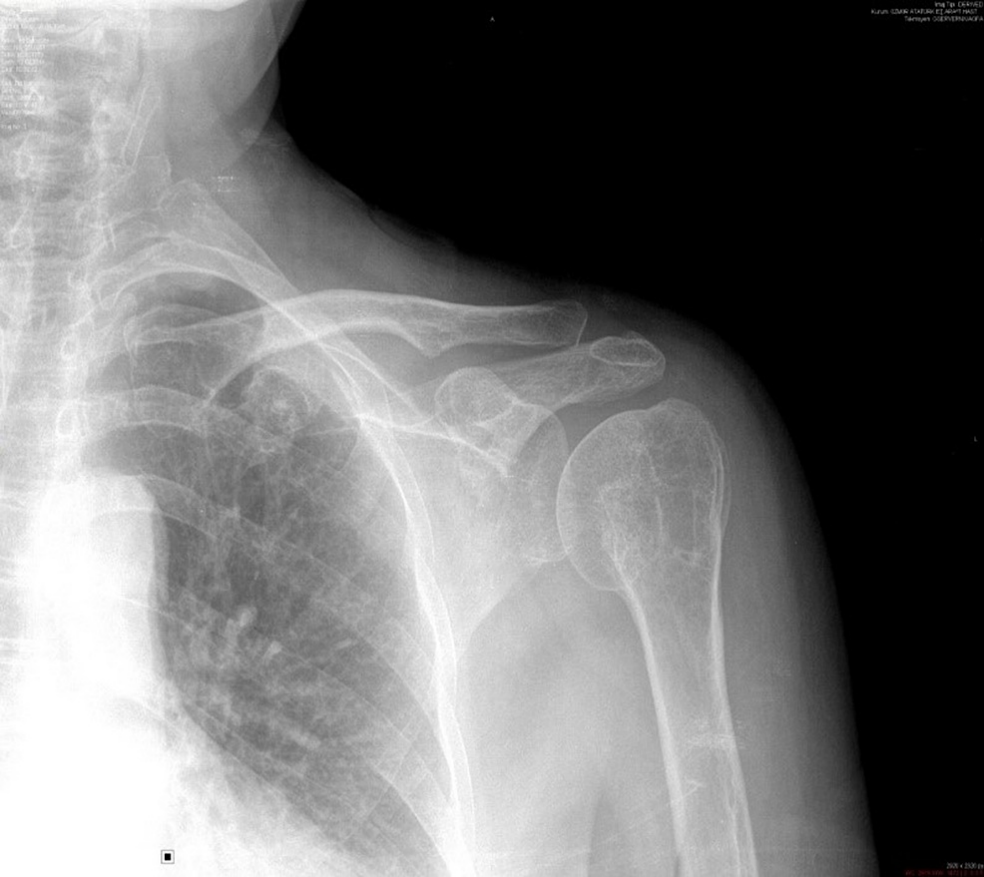
X-ray after conservative follow-up, full union achieved.

For patients in group III, after four weeks of Velpeau bandage resting, active wrist and elbow exercises as well as passive shoulder exercises were performed. Active-assisted exercises were started in the sixth week, and strengthening exercises were started in the eighth week (Figures 5, 6).

Statistical analysis

For statistical analysis, IBM SPSS Statistics Software version 22.0 (IBM Corp., Armonk, NY) was used to analyse the data. The distribution of data was evaluated with the Shapiro Wilk test. For normally distributed data mean values ±SD and for non-normally data median values [Interquartile range (IQR): 25th percentile-75th percentile] were presented. For quantitative data that were not normally distributed Kruskal-Wallis test and post hoc Dunn’s test were used. One-Way ANOVA test and post hoc Bonferroni test were used for normally distributed data. The Chi-square test was used to compare categorical variables. Qualitative data were presented as percentages. For all tests, p < 0.05 was considered statistically significant.

## Results

Thirty-two patients were female, and 15 were male. The oldest was 90 years old, the youngest was 50 years old, and the mean age was 71.5 ± 10.7 years. Thirty-four patients were Type III according to the Neer Classification, and 13 patients were Type IV (Table 1). There was no significant difference in age (p = 0.055), gender (p = 0.555), fracture type (p = 0.410) and follow-up period (p = 0.271) between groups.

**Table 1:** Distribution of fracture types and demographic data between groups. OS: Locking plate osteosynthesis, HA: Hemiarthroplasty, CT: Conservative treatment.

Treatment Groups					
	OS (n = 18)	HA (n = 15)	CT (n = 14)	P	
Mean Age	69.5 ± 11.5	68.5 ± 11.3	77.1 ± 6.5	0.055	
Gender F/M	12 (66.7%) / 6 (33.3%)	9 (60%) / 6 (40%)	11 (78.6%) / 3 (21.4%)	0.555	
Neer Classification Type III / Type IV	12 (66.7%) / 6 (33.3%)	10 (66.7%) / 5 (33.3%)	12 (85.7%) / 2 (14.3%)	0.410	
Mean Follow-up Period (Months)	24.2 (19.7-37.9)	42.3 (20.9-53.8)	33.1 (24.6-38.9)	0.271	

When the scores of the patients who had undergone locking plate osteosynthesis were evaluated, the mean Constant score was 71.6 ± 16.2. Three of the patients had excellent results, four had good results, four had moderate results, and seven had poor results. The average DASH score was 12.1 (5.2-24.2), and the average ASES score was 77.5 (50.8-96.6). When the scores of the patients who had undergone hemiarthroplasty were evaluated, the mean Constant score was 49.7 ± 11.8 and all 15 had poor results. The average DASH score was 23.3 (14.6-36.2), and the average ASES score was 54.9 (41.6-78.3). When the scores of the patients in group III were evaluated, the mean Constant score was 69.6 ± 19.2. One of the patients had excellent results, six had good results, three had moderate results, and four had poor results. The average DASH score was 16.4 (12.5-36.7), and the average ASES score was 76.6 (45.4-87.9).

When the data were examined, the data that were statistically significant as a result of comparing the three groups were evaluated with post hoc tests (Table 2).

**Table 2:** Evaluation of functional results by treatment type. p < 0.05 considered significant. IQR: Interquartile range, DASH: Disabilities of the arm, shoulder and hand, ASES: American Shoulder and Elbow Society, OS: Locking plate osteosynthesis, HA: Hemiarthroplasty, CT: Conservative treatment.

	Evaluation of Functional Results			
		Groups	Mean ± SD / Median (IQR)	P
DASH Score		CT	16.4 (12.5-36.7)	0.032
		HA	23.3 (14.6-36.2)	
		OS	12.1 (5.2-24.2)	
Constant Score		CT	69.6 ± 19.2	0.001
		HA	49.7 ± 11.8	
		OS	71.6 ± 16.2	
ASES Score		CT	76.6 (45.4-87.9)	0.090
		HA	54.9 (41.6-78.3)	
		OS	77.5 (50.8-96.6)	
Forward Elevation		CT	99.6 ± 31.0	<0.001
		HA	61.0 ± 22.9	
		OS	106.1 ± 34.3	
Abduction		CT	97.1 ± 24.9	<0.001
		HA	55.0 ± 18.2	
		OS	101.7 ± 37.9	
İnternal Rotation		CT	60 (30.0-60.0)	0.022
		HA	30 (30.0-45.0)	
		OS	52.5 (30.0-60.0)	
External Rotation		CT	60 (30.0-67.5)	0.048
		HA	30 (10.0-60.0)	
		OS	60 (30.0-75.0)	
Extension		CT	45 (30.0-45.0)	0.074
		HA	30 (30.0-45.0)	
		OS	45 (30.0-45.0)	

When constant and DASH scores for group I and group III were examined, no statistically significant difference was found (Constant p > 0.999, DASH p = 0.285). There were also no statistically significant differences in shoulder flexion (p > 0.999), abduction (p > 0.999), internal rotation (p > 0.999), external rotation (p > 0.999) between the two groups.

Group I and group II were compared using the same method. Constant scores (p = 0.001), DASH scores (p = 0.032), shoulder flexion (p < 0.001), shoulder abduction (p < 0.001) and internal rotation (p = 0.044) were statistically significantly different. There were no statistically significant differences in external rotation (p = 0.106).

When group II and group III were compared with the same method, there was a statistically significant difference in Constant score (p = 0.005), shoulder flexion (p = 0.004) and shoulder abduction (p = 0.001). There were no statistically significant differences in the DASH score (p > 0.999), shoulder external rotation (p = 0.089) and shoulder internal rotation (p = 0.053).

## Discussion

Campbell [18] argued that impacted fractures in elderly patients should be treated with conservative methods and that even radiographically serious malpositions can be functionally tolerated. Various fixation methods have been developed for surgical treatment, but the gold standard method cannot be specified due to complication rates of up to 50% [11,18,19].

In a meta-analysis, Mao et al. [20] compared surgery and conservative treatment in Neer Type III and Type IV fractures. Among the surgical treatments in their study, they evaluated intramedullary nailing, locking plate, minimally invasive plate application and arthroplasty together. They evaluated the patients with Constant scoring and reported that there was no functional difference between conservative and surgical treatments [20].

In studies comparing locking plate surgery and conservative treatment for three- and four-part fractures, no difference was observed in the results of elderly patients in the one-year follow-up period [21]. In the current study, mean scores and mean ranges of motion were evaluated, and the locking plate osteosynthesis group was found to be superior; however, there was no statistically significant difference compared to the conservative treatment group. We think that the higher ASA scores of patients in group III and its effect on physical activity performance contributed significantly to this conclusion.

For the locking plate osteosynthesis and conservative treatment groups, nonunion occurred in three of 32 patients. In their study, Iyengar et al. reviewed 12 studies involving 650 patients and stated that 98% union was achieved in patients who were followed up conservatively, and the complication rate was 13% [22]. Charalambous et al. reported that five of 25 patients treated with a locking plate required revision due to implant failure and non-union [23]. Screw migration was one of the most important complications in fixation with a locking plate. Lill et al. reported a complication rate of 17% in their published series of screw migration complications [24]. In the current study, the union rate was 90.6%, and the screw migration complication rate was 16.6%. Reoperations were required for implant removal due to screw migration in three patients, subacromial impingement in three patients, deep tissue infection in one patient and avascular necrosis in one patient. Functional scores and range of motion values ​​for the locking plate osteosynthesis group are compatible with the literature, and the results are similar to the conservative treatment group [25-26].

Kraulis and Hunter reported that only two patients out of 11 treated with hemiarthroplasty showed satisfactory results [27,28]. In a randomised controlled study of elderly patients by Olerud et al., they stated that there was no difference between hemiarthroplasty and conservative treatment in four-part proximal humerus fractures in terms of the range of motion after two years of follow-up. However, they reported that patients with hemiarthroplasty had significantly fewer complaints of pain [29]. For hemiarthroplasty, it is important to note that complications can be minimised by paying attention to the surgical details [30-32]. The most common cause of failure in hemiarthroplasty is poorly fixated tubercules; humerus length, appropriate retroversion and tubercles should be restored properly [32]. In the current study, four of the 15 hemiarthroplasty patients had insufficient tubercular fixation in their early postoperative radiographs.

Green et al. evaluated 22 patients who underwent hemiarthroplasty with ASES scores. They found the average forward flexion to be 100° and external rotation to be 30° [33]. In the current study, the mean forward flexion was 61°, and the external rotation was 35° in the hemiarthroplasty group.

Although hemiarthroplasty gives successful results in terms of subjective criteria, it does not provide the expected improvement in terms of functional results. In addition, we believe that it is more appropriate to use hemiarthroplasty in multipart fractures, including anatomical neck fractures, and in patients with high probability of developing avascular necrosis according to Hertel’s criteria [34]. In terms of surgical treatment, we found that locking plate osteosynthesis has better clinical results than hemiarthroplasty. However, in patients undergoing locking plate osteosynthesis, the need for reoperation due to implant removal and loss of reduction can be considered a deterrent. As it is known, various complications such as loss of fracture reduction, nonunion, avascular necrosis can be seen after plate fixation of comminuted proximal humerus fractures in elderly and osteoporotic patients. One of the advantages of hemiarthroplasty is the elimination of such complications that may develop related to bone union.

The functional results of the locking plate osteosynthesis and conservative treatment groups were statistically the same. However, we think that conservative treatment should be evaluated in the foreground in patients with advanced age, additional morbidity and when surgery poses a high risk.

There are several limitations to this study. It is seen that there is a difference between the physical performance capacities of the patients between the groups. In terms of functional outcomes, it is obvious that patients in group III with high ASA scores have lower scores. The number of patients was insufficient, the follow-up period was relatively short, the operations were performed by different surgical teams, and the study was retrospective.

## Conclusions

Hemiarthroplasty should not be considered a priority in surgical planning in Neer three-part and four-part proximal humerus fractures. We think that conservative treatment is superior for patients with additional morbidity and advanced age, but in patients who are younger and can tolerate the surgical procedure, the priority should be locking plate osteosynthesis.
